# The impact of the digital divide on residents' healthcare consumption inequality: evidence from CFPS in China

**DOI:** 10.3389/fpubh.2025.1699498

**Published:** 2026-01-14

**Authors:** Jikai Yang, Bingquan Luo, Liangping Hu

**Affiliations:** 1 School of Sports Management and Communication Capital University of Physical Education and Sports, Beijing, China; 2 School of Leisure and Social Sports, Capital University of Physical Education and Sports, Beijing, China

**Keywords:** consumption inequality, digital divide, healthcare, healthcare consumption inequality, residents' consumption

## Abstract

**Introduction:**

To explore the impact of the digital divide on residents' healthcare consumption inequality, reveal its underlying transmission mechanisms and heterogeneous effects, address the gap in existing literature that focuses on macro-level health inequality but neglects micro-level disparities, and provide theoretical support and actionable policy recommendations for reducing global healthcare consumption inequality amid digital transformation.

**Methods:**

Multi-wave data from the China Family Panel Studies (CFPS) were used as the analytical sample. The Kakwani index was utilized to measure both healthcare consumption inequality and the digital divide. A two-way fixed-effects model and a mediation model were applied to examine the impact of the digital divide on residents' healthcare consumption inequality.

**Results:**

(1) The digital divide significantly exacerbates residents' healthcare consumption inequality, with a regression coefficient of 0.0523 (*p* < 0.01) after controlling for individual, household, and regional factors. (2) Income inequality and credit constraints serve as key mediating pathways, accounting for 48.37% and 43.99% of the total effect, respectively. (3) Heterogeneous effects are evident: the impact of the digital divide is weaker in rural regions and economically underdeveloped areas, but stronger among residents with low education levels and high-income households.

**Conclusion:**

Theoretically, this study extends health inequality research from the macro-level to the micro-level by linking the digital divide to healthcare consumption disparities, and validates the applicability of Keynesian consumption theory and the life-cycle hypothesis in the digital era. Furthermore, the findings align with the United Nations' Sustainable Development Goals (SDGs 3 and 10): targeted interventions can mitigate the adverse effects of the digital divide. For developing countries, this study offers a strategic framework to balance digital healthcare advancement with equity, preventing technological exclusion from exacerbating healthcare disparities.

## 1 Introduction

Inequality has existed worldwide for decades. Pursuing equality is one of the goals of the United Nations' 2030 Agenda for Sustainable Development Goals [SDGs; (1)]. Health inequality refers to avoidable and unfair differences in health outcomes across populations (2, 3) and is a central focus of Sustainable Development Goals (SDGs) 3 and 10 (4). Over the years, policymakers, researchers, and public health practitioners have conducted extensive macro-level studies on health inequalities, identifying factors such as ethnicity (5), occupation (6), income (7), intergenerational transmission (8), and major public health events (9) as key determinants. Although health inequality research has become systematic, it has largely focused on macro-level factors, with limited exploration of micro-level determinants (10).

Healthcare is a key determinant of residents‘ health (11), and healthcare expenditures influence the quality and accessibility of services individuals can obtain. In the aftermath of the COVID-19 pandemic, healthcare consumption has become an increasingly important component of Chinese residents' overall consumption. According to data from the National Bureau of Statistics of China, per capita healthcare consumption among Chinese residents has risen steadily in recent years, from 1,902 yuan in 2018 to 2,547 yuan in 2024, representing approximately 9.02% of total per capita consumption expenditures in that year. Although demand for healthcare is rising, existing research indicates that inequalities in healthcare consumption persist across social strata (12), and may further widen amid economic growth and rising living costs (13).

With the advent of the Fourth Industrial Revolution, the development of digital technology has driven the digital transformation of healthcare consumption (14). Digital technology can integrate resources to provide residents with the latest health information and enhance the accessibility of healthcare services (15). Examples include telehealth services and internet healthcare, which utilize the internet as a platform and technological tool to deliver various forms of healthcare services such as health education, medical information inquiries, electronic health records, disease risk assessments, online disease consultations, electronic prescriptions, remote consultations, and remote treatment and rehabilitation (16). This helps address the imbalance in China's healthcare resources and the growing demand for healthcare services (17), making it a healthcare development model actively promoted and supported by National Health Commission of China (18).

In this context, the concept of Digital Health Equality (DHE) emerges. DHE is a multi-layered social ecosystem concept rooted in the equal opportunity for every individual to achieve the highest attainable standard of health through digital health resources and services (19). It is essential for achieving the goals of healthcare system transformation, including optimizing patient experience, improving population health, protecting the well-being of healthcare workers, reducing costs, and advancing health equity. Current research on digital health equity predominantly focuses on cultural and gender perspectives, with few studies addressing economic disparities in healthcare consumption in the digital era. Furthermore, some studies suggest that digital technologies may simultaneously widen existing digital divides or generate new forms of exclusion, potentially exacerbating inequalities in healthcare consumption across population groups. Together, these gaps highlight the importance of the present study.

While digital technology has introduced new forms of healthcare consumption for residents (20), it has also given rise to a digital divide (21). The Organization for Economic Co-operation and Development (OECD) defines the digital divide as “the gap in opportunities between individuals, households, businesses, or geographic areas at different socioeconomic levels to access information and communication technologies and to participate in various online activities.” The “14th Five-Year Plan for Digital Economic Development” states that the digital divide across industries, regions, and demographic groups in China has not been effectively bridged and may be widening. A review of existing research shows that scholars have confirmed from various perspectives—such as technological innovation (22), urban-rural disparities (23), and public health events (24)—that the digital divide exacerbates economic inequality among residents and contributes to disparities in social and economic development (25).

Existing research on the digital divide and health equity has primarily focused on developed countries in Europe and North America, with few studies conducted in developing countries. Furthermore, although some studies have acknowledged the mediating role of socioeconomic factors in the relationship between the digital divide and population health, they have relied exclusively on macro-level analyses. To date, no research has examined the impact of the digital divide on disparities in healthcare consumption. To address this gap, this study uses data from the China Family Panel Studies (CFPS) to examine the relationship between the digital divide and inequality in residents' healthcare consumption, aiming to provide quantitative evidence to inform policy in digital technology and healthcare.

## 2 Research hypotheses

### 2.1 The impact of the digital divide on inequality in healthcare consumption among residents

The digital divide reduces access to healthcare services and exacerbates healthcare consumption inequality (26, 27). Income disparities have been identified as a key mediator in the relationship between the digital divide and inequalities in residents' healthcare consumption. Existing studies have examined the mediating role of income disparities in this relationship across various contexts, including energy poverty (28), technological innovation (19), and urban–rural divides (23). However, these studies have largely overlooked the Chinese context, particularly the role of informal institutions such as social capital, which shape access to financial resources and may further mediate the link between digital exclusion and health inequality. This study argues that the relationship between the digital divide and healthcare consumption inequality warrants further analysis from three dimensions: access to digital devices, digital technology use, and engagement with digital content.

First, in terms of access to digital devices, ownership is a prerequisite for participating in modern digital healthcare. Individuals with access to digital devices can more conveniently obtain healthcare services and utilize digital platforms (29). Populations lacking smartphones or stable internet access—such as the older adult, rural residents, and low-income groups—cannot utilize digital healthcare services such as online appointment booking, telemedicine consultations, health information searches, or e-pharmacies, and must rely on traditional offline channels (30). However, offline channels face challenges such as uneven resource distribution, high time costs, and information asymmetry, thereby exacerbating healthcare consumption inequality.

Second, in terms of digital technology use, some residents with access to digital tools may still be excluded from the benefits of digital healthcare due to limited digital literacy or inability to navigate health applications (31). Specifically, individuals with low digital skills may avoid using digital healthcare services due to operational difficulties or misinterpretation of information. In contrast, those with high digital literacy can effectively use digital tools to reduce unnecessary visits, choose cost-effective care options, and optimize healthcare utilization—thereby widening disparities in healthcare consumption (32).

Third, with regard to digital health information access, healthcare information in the digital age is fragmented and often shaped by algorithmic filtering, leading to unequal exposure. Individuals with higher digital literacy can effectively use search and filtering tools to access accurate health information and make informed healthcare decisions (33). In contrast, individuals with lower digital literacy are more likely to encounter misinformation or low-quality content, often trapped in filter bubbles—leading to biased healthcare decisions and widened consumption inequalities (34).

Accordingly, the first hypothesis is proposed as follows:

H1: The digital divide will widen inequalities in healthcare consumption among residents.

#### 2.1.1 The transmission mechanism of the digital divide on inequality in healthcare consumption among residents

Currently, income inequality among Chinese residents is significant and growing, and it is a major driver of consumption disparities (35). The combined and interactive effects of income inequality and the digital divide may further exacerbate these disparities (36). According to the Keynesian consumption function, income is a key determinant of residents' healthcare consumption (37). Therefore, income inequality will contribute to unequal healthcare consumption. Since the 18th National Congress of the Communist Party of China, China's digital economy has expanded rapidly, and digital transformation has profoundly influenced various aspects of residents' lives. However, the resulting digital divide has become a significant factor in widening income inequality. From an individual-level perspective, digitally disadvantaged individuals face greater challenges in mastering digital technologies. Existing research confirms that the skill-biased nature of the labor market favors high-tech, highly skilled workers. Consequently, the digital divide limits income opportunities for disadvantaged groups, contributing to healthcare consumption inequality (38). From a regional perspective, the uneven development of the digital economy has resulted in significant disparities in digital infrastructure and technology adoption across regions (39). As a result, factor flows between regions remain asymmetric. Regions with more advanced digital economies can leverage their advantages to attract talent, capital, and high-tech industries, further widening regional disparities and exacerbating income inequality—thereby contributing to inequalities in healthcare consumption.

The life-cycle theory of consumption posits that borrowing enables individuals to smooth consumption over time (40). Credit constraints refer to the barriers individuals face in accessing loans, limiting their ability to make optimal consumption decisions (41). As a result, individuals may reduce current consumption to buffer against future uncertainties, making credit constraints a key determinant of consumption levels (42). The rapid development of digital technology has significantly alleviated residents' credit constraints. Innovations such as digital inclusive finance and fintech platforms have disrupted traditional lending practices (43). Financial institutions now use big data to collect and analyze consumer credit information, enabling more accurate risk assessment, reduced information asymmetry, and eased formal credit constraints (44). Additionally, digital platforms expand users' social networks, strengthen social capital, and facilitate access to informal credit (45). However, digitally disadvantaged individuals—due to limited device access and low digital literacy—may be excluded from digital financial platforms, preventing access to both formal and informal credit channels. Consequently, the digital divide may intensify credit constraints, undermine intertemporal consumption smoothing, reduce healthcare spending, and ultimately exacerbate healthcare consumption inequality (46).

Accordingly, the second hypothesis is proposed as follows:

H2: In the process of digital divide affecting inequality in healthcare consumption among residents, income inequality and credit constraints play an mediating role.

**Figure 1:**
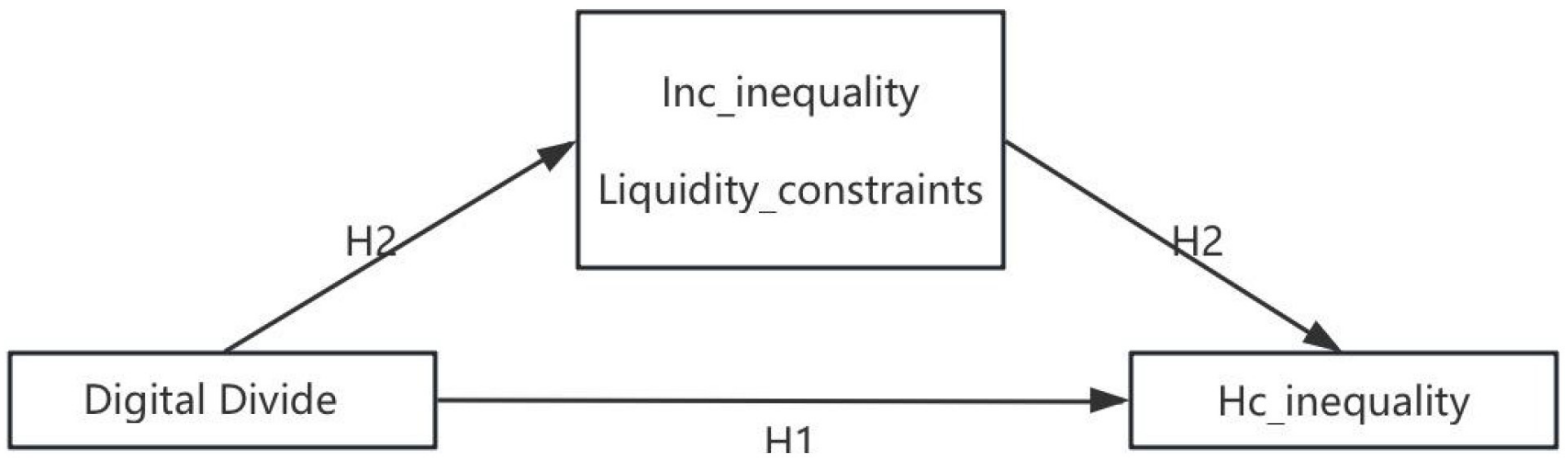
Descriptive statistics.

The mechanism of action in this study is shown in Figure 1.

## 3 Methods and data

### 3.1 Approach

This paper uses the Kakwani index to measure the healthcare consumption inequality index among residents. The Kakwani Index is a classic measure of relative deprivation. Its core logic lies in quantifying the overall level of “relative deprivation” by comparing individuals with higher-consumption groups within the same region. Its advantage is that it not only focuses on the dispersion of consumption distribution but also reflects an individual's relative position within a group, better aligning with the “class disparity” inherent in healthcare consumption inequality. This study calculates the index using provinces as the reference group. Higher values indicate greater inequality in healthcare consumption distribution. Compared to the Gini coefficient, it emphasizes “relative deprivation” rather than absolute disparities. Combining both measures enhances measurement comprehensiveness.

### 3.2 Data sources

The data used in this study are sourced from the China Family Panel Studies (CFPS) conducted by Peking University. The CFPS is a national, comprehensive social tracking survey project that collects information on communities, households, and individuals through a PPS sampling method. The CFPS survey began in 2010 and is conducted every 2 years, covering three levels: individuals, households, and communities. The survey targets all household members and covers approximately 95% of the national population.

Starting in 2016, the China Family Panel Studies (CFPS) questionnaire was revised to include more detailed questions on digital device usage, explicitly distinguishing between internet access via computers and via mobile phones. This refinement is especially relevant in an era of widespread smartphone adoption. Moreover, post-2016 surveys exhibit lower rates of missing data on digital technology use, thereby improving data quality and strengthening the validity of empirical analyses. Therefore, this study utilizes data from four waves: 2016, 2018, 2020, and 2022. By merging household head information with household information, an unbalanced panel dataset was constructed. After removing missing and outlier values, a total of 35,970 samples were obtained for analysis.

### 3.3 Variables

#### 3.3.1 Dependent variable

The dependent variable in this study is healthcare consumption inequality among residents (Hc_inequality). To measure this inequality, we employ the relative deprivation index (RDI), which assesses individuals' perceived deprivation by comparing their healthcare spending to that of higher-spending peers within the same provincial reference group. Responses to the CFPS survey question—“In the past year, how much did your household spend on healthcare expenses (including fitness and exercise, as well as the purchase of related products, equipment, and health supplements, etc.)?”—serve as the measure of individual healthcare consumption. At the aggregate level, healthcare consumption inequality is quantified using the Kakwani index. The Gini coefficient is also calculated to conduct robustness checks. Higher values of both indices indicate greater inequality in healthcare consumption distribution, while lower values suggest a more equitable distribution. Specifically, assuming that reference group X has n observations, the average healthcare consumption is, and the healthcare consumption vector of individuals within the group is X = x_1_, x_2_, x_3_, ……, x_n_, while x_1_ ≤ x_2_ ≤ x_3_ ≤ …… ≤ x_n_, let the average healthcare consumption of samples within the group whose healthcare consumption exceeds that of individual i be denoted as $\mu_{{\text{x}}_{\text{i}}}^{+}$, and let the proportion of observations whose healthcare consumption exceeds $\mu_{{\text{x}}_{\text{i}}}^{+}$ be denoted as $\gamma_{{\text{x}}_{\text{i}}}^{+}$. The specific formula is as follows:


$$\begin{array}{l} Kakwani\left(x_{1},x_{n}\right)=\frac{1}{n\mu_{x}}\overset{n}{\underset{j=i+1}{\sum }}\left({X_{j}-X}_{i}\right)=\gamma_{x_{i}}^{+}\left[\left(\mu_{x_{i}}^{+}-x_{i}\right)/\mu_{x}\right] \end{array}$$ (1)

#### 3.3.2 Independent variable

The independent variable in this study is the digital divide. Using a single indicator to measure the digital divide may fail to capture the multifaceted impact of digital technology on healthcare consumption. Therefore, this study examines the digital divide along two dimensions—accessibility and usage of digital technology—by integrating the CFPS questionnaire design with data availability. Simultaneously, based on the OECD's definition of the digital divide (a dual-core framework of access and usage), we examine the digital divide from two dimensions: the accessibility of digital technologies and their usage patterns. Accessibility serves as the prerequisite for participation in digital healthcare, while usage reflects the practical application of digital skills. Together, these dimensions encompass the core essence of the digital divide. Moreover, data can be directly extracted from multiple waves of CFPS, ensuring the feasibility and validity of the measurement.

For accessibility, responses to the questions “Do you use mobile internet?” and “Do you use a computer to access the internet?” are used as proxy indicators. Digital technology usage reflects individuals' reliance on digital tools in work, study, business, social interactions, and entertainment. Given changes in the CFPS questionnaire over time, this study follows established methodologies by using the frequency and perceived importance of internet use across these five domains as proxy measures. Principal component analysis (PCA) is applied to reduce dimensionality, generating a composite digitalization index, which is then standardized. To account for regional disparities in digital development, the Kakwani index is used to measure inequality in the digitalization index, with individuals within the same province serving as the reference group. This yields the digital divide index.

#### 3.3.3 Control variables

To minimize potential bias from omitted variables in the statistical model, this study includes control variables at three levels: individual, household, and regional. Individual-level controls include age, gender, household registration, years of education, marital status, employment status, health status, and medical insurance status. Household-level controls include housing conditions, household size, household debt, total household income, and net household assets. Regional-level controls include regional economic development and regional consumer market size.

### 3.4 Modeling design

To empirically analyze the impact of the digital divide on inequality in residents' healthcare consumption, this study constructs the following model:


$$\begin{array}{l} {\text{Hc\_inequality}}_{\text{ict}}=\alpha +\beta Divide_{ict}+\gamma X_{ict}+\eta_{c}+\vartheta_{t}+\varepsilon_{ict} \end{array}$$ (2)

In the model (2), Hc_inequality_ict_ represents the level of inequality in healthcare consumption for resident i in region c at time t, Divide_ict_ is the core explanatory variable, the digital divide index for residents, X_ict_ is the control variable, including individual variables represented by the head of household, household variables, and regional economic variables, α is the intercept term, β and γ are the coefficients to be estimated, η_c_ is the province fixed effect, ϑ_t_ is the year fixed effect, and ε_ict_ is the random disturbance term. To avoid the potential impact of correlation on the results, standard errors are clustered at the provincial level.

To verify the transmission mechanism of the digital divide on inequality in residents' healthcare consumption, this paper uses a stepwise regression model to examine the mediating effect. The following is the stepwise regression model (Equations 3–5):


$$\begin{array}{lll} {\text{Hc \_ inequality}}_{ict} & = & \alpha_{1}+\beta_{1}Divide_{ict}+\gamma_{1}X_{ict}+\eta_{c}+\vartheta_{t}+\varepsilon_{ict} \end{array}$$ (3)

$$\begin{array}{lll} \;M_{ict} & = & \alpha_{2}+\beta_{2}Divide_{ict}+\gamma_{2}X_{ict}+\eta_{c}+\vartheta_{t}+\varepsilon_{ict} \end{array}$$ (4)

$$\begin{array}{lll} {\text{Hc \_ inequality}}_{ict} & = & \alpha_{3}+\beta_{3}Divide_{ict}+{\lambda_{3}M}_{ict}+\gamma_{1}X_{ict} \end{array}$$ (5)

$$\begin{array}{l} \;+\eta_{c}+\vartheta_{t}+\varepsilon_{ict} \end{array}$$

## 4 Results

### 4.1 Descriptive statistics

Table 1 presents the descriptive statistics for the key variables in this study. The mean value of the critical dependent variable “Hc_inequality” is 0.9325. According to data from the National Bureau of Statistics of China, an inequality coefficient exceeding 0.40 indicates extreme inequality in healthcare expenditures within China. Measured by the Gini coefficient, the mean value is 0.9369, consistent with the Kakwani index results, demonstrating robustness. The mean value of the key independent variable “Divide” is 0.5183, also exceeding 0.40.

**Table 1:** Descriptive statistics.

**Variable**	**Definition**	**Sample**	**Mean**	**S.D**.	**Min**	**Max**
Hc_inequality	The degree of inequality in residents' healthcare consumption calculated computed the Kakwani index	35,970	0.9325	0.1829	0	1
Hc_inequality(Gini)	The degree of inequality in residents' healthcare consumption calculated computed the Gini index	35,970	0.9369	0.1412	0	1
Divide	Digital divide for residents calculated computed the Kakwani index	35,970	0.5183	0.4213	0	1
Age	Actual interviewee age at the time of the survey, in years	35,970	47.3712	13.4475	18	93
Age^2^/100	The quadratic term of the respondent's age divided by 100	35,970	24.2486	13.0089	3.2400	86.4900
Gender	Female = 0, Male = 1	35,970	0.5406	0.4984	0	1
Registration	Agricultural household registration = 0, non-agricultural household registration = 1	35,970	0.2398	0.4269	0	1
Education levels	Years of schooling among residents	35,970	8.2221	4.9487	0	22
Marital status	Unmarried, divorced, widowed, etc = 0, married = 1	35,970	0.8882	0.3151	0	1
Employment status	Unemployed = 0, employed = 1	35,970	0.8096	0.3927	0	1
Health status	Self-rated health on 1-5 scale; higher value denotes better health	35,970	3.0323	1.1912	1	5
Insurance status	uninsured = 0, insured = 1	35,970	0.9243	0.2646	0	1
Housing status	No housing = 0, have housing = 1	35,970	0.7770	0.4162	0	1
Family size	Total population of the family; unit: person	35,970	3.7469	1.8942	1	19
Household debt	Have household debts = 1; no = 0	35,970	0.2036	0.4027	0	1
Household income	Total household income; based on 2010 as the baseline, take logarithm processing	35,970	10.8086	1.0831	0	16.3898
Household assets	Total household assets; based on 2010 as the baseline, take logarithm processing	35,970	11.8964	2.7629	0	17.7700
Regional economic development	Per capita GDP by province, take logarithm processing	35,970	10.9297	0.4048	10.2182	12.1547
Regional consumer market size	Ratio of provincial retail sales of consumer goods to GDP	35,970	0.3956	0.0553	0.1827	0.5384

### 4.2 Benchmarking results

Based on the Hausman test results, a fixed-effects model is employed to estimate the effect of the digital divide on residents' healthcare consumption inequality. The estimation results for model (2) are presented in Table 2. In Column (1), which includes only the digital divide variable, a one-unit increase in the digital divide is associated with a 0.092 rise in the healthcare consumption inequality index (*p* < 0.01), indicating a strong positive relationship. In Column (2), after controlling for individual, household, and regional factors, the digital divide remains positively and significantly associated with healthcare consumption inequality. Additionally, the model's fit improves, as reflected in a higher pseudo R^2^. These findings support Hypothesis H1.

**Table 2:** Benchmark regressions results.

**Variable**	**(1)**	**(2)**
	**Hc_inequality**	
Divide	0.0915^***^	0.0523^***^
	(0.0068)	(0.0052)
Age		0.0027^***^
		(0.0005)
Age^2^/100		−0.0036^***^
		(0.0005)
Gender		0.0115^***^
		(0.0026)
Registration		−0.0405^***^
		(0.0058)
Education levels		−0.0038^***^
		(0.0005)
Marital status		0.0080^**^
		(0.0034)
Employment status		−0.0028
		(0.0025)
Health status		−0.0028^***^
		(0.0010)
Insurance status		−0.0008
		(0.0030)
Housing status		−0.0062^*^
		(0.0031)
Family size		0.0013^*^
		(0.0006)
Household debt		−0.0163^***^
		(0.0033)
Household income		−0.0201^***^
		(0.0019)
Household assets		−0.0043^***^
		(0.0006)
Regional economic development		0.0534
		(0.0446)
Regional consumer market size		0.0377
		(0.1258)
Constant	0.8851^***^	0.5756
	(0.0035)	(0.5249)
Area FE	YES	YES
Year FE	YES	YES
Obs	35,970	35,970
Adj R^2^	0.0725	0.1201

^*^, ^**^, ^***^ indicates statistical significance at the 1%, 5%, 10% level. The values in parentheses represent the cluster standard errors, with the same notation applied throughout.

The coefficient of Divide (0.0523) means a one-unit rise in the digital divide index increases the healthcare consumption inequality index by 0.0523 units, ceteris paribus.

This is because, in the era of “information explosion”, consumers rely on digital platforms throughout the healthcare consumption process—including information search, product evaluation, payment, and post-purchase rights enforcement. However, the digital divide renders critical healthcare information inaccessible or invisible to digitally disadvantaged groups (47). These individuals face information asymmetry in healthcare consumption, leading to relative deprivation and exacerbating healthcare consumption inequality (48). Specifically, those constrained by the digital access divide may be unable to obtain timely healthcare information through online platform notifications or other digital channels (33), while those affected by the digital usage divide may lack the skills to navigate health insurance information or apply for online consumption vouchers, resulting in unequal consumption outcomes (49).

### 4.3 Robustness test

To further assess the robustness of the benchmark regression results, a series of robustness checks were conducted, including outlier removal, partial sample removal, alternative measures of the explanatory variable, and instrumental variable estimation.

#### 4.3.1 Outlier removal

In order to eliminate the influence of certain special samples on the overall research conclusions, the explained variables were subjected to two-sided trimming at the 1% and 3% percentiles, and the regression was performed again. The results are shown in columns (1) and (2) of Table 3.

**Table 3:** Regression results of robustness testing.

**Variable**	**(1)**	**(2)**	**(3)**	**(4)**	**(5)**
	**Hc_inequality**				
Divide	0.0516^***^	0.0481^***^	0.0503^***^	0.0513^***^	0.0420^***^
	(0.0052)	(0.0049)	(0.0053)	(0.0048)	(0.0041)
Controls	YES	YES	YES	YES	YES
Area FE	YES	YES	YES	YES	YES
Year FE	YES	YES	YES	YES	YES
Obs	35,970	35,970	32,292	33,397	35,970
Adj R^2^	0.1188	0.1195	0.1231	0.1181	0.1184

Due to the exclusion of certain variables, the number of observations has changed. ^*^, ^**^, ^***^ indicates statistical significance at the 1%, 5%, 10% level. The values in parentheses represent the cluster standard errors, with the same notation applied throughout.

#### 4.3.2 Partial sample removal

First, to avoid the impact of factors such as schooling and retirement on healthcare consumption, the age range was restricted to 22–65 years for the analysis. Second, to avoid the influence of the economic development advantages of developed regions on the research results, the four municipalities were excluded, and the analysis was repeated. The results are shown in columns (3) and (4) of Table 3.

#### 4.3.3 Changing the measurement method of the dependent variable

The Gini coefficient was used to reconstruct the explanatory variable Hc_inequality, and the results are shown in column (5) of Table 3.

#### 4.3.4 Instrumental variable methods

Although this study included as many observable control variables as possible and employed two-way fixed effects for empirical analysis, endogeneity issues may still exist. Therefore, an instrumental variable approach was used to test for endogeneity and ensure the reliability of the research results. Following existing research, monthly telecommunications expenses (IV_tel) are employed as an instrumental variable for the digital divide to address endogeneity concerns. Telecommunications expenses can characterize residents' internet usage costs, capture a negative association with the digital divide, and are expected to have no direct impact on healthcare consumption inequality. This satisfies the relevance condition (strong correlation with the endogenous explanatory variable) and the exclusion restriction (no direct effect on the dependent variable), making it a valid instrument.

The results are reported in Table 4. Based on the results of the first-stage regression in column (1), “monthly telecommunications expenses” is significantly negatively correlated with the digital divide index and is a suitable instrumental variable. The Kleibergen-Paap Wald F-value is 51.0320, which is greater than the critical value at the 10% significance level in Stock-Yogo, indicating that the instrumental variable passes the weak instrument test. Column (2) of Table 4 reports the results of the second-stage regression. The digital divide still has a significant widening effect on inequality in residents' healthcare consumption, consistent with the results of previous studies, confirming its reliability.

**Table 4:** Regression test results using instrumental variables.

**Variable**	**(1)**	**(2)**	**(3)**
	**Divide**	**Hc_inequality**	**Hc_inequality**
IV_tel	−0.0001^***^		
	(0.0000)		
Divide		0.7890^***^	0.0450^***^
		(0.0672)	(0.0056)
Controls	YES	YES	YES
Area FE	YES	YES	YES
Year FE	YES	YES	YES
Obs	35,970	35,970	13,386
Adj R^2^	–	–	0.1177
KP W F	51.0320		–

Due to the re-matching process, the number of observations has changed. ^*^, ^**^, ^***^ indicates statistical significance at the 1%, 5%, 10% level. The values in parentheses represent the cluster standard errors, with the same notation applied throughout.

In summary, the robustness checks support the baseline findings, confirming that the digital divide exacerbates inequality in residents' healthcare consumption.

#### 4.3.5 Reverse causality test

This study also carries the risk of reverse causality. On one hand, healthcare expenditure inequality may indirectly influence the digital divide through income constraints, as households with higher healthcare spending may reduce investment in digital devices and internet access, leading to insufficient digital connectivity. On the other hand, the underlying class disparities (such as education and income) driving healthcare expenditure inequality may simultaneously contribute to the digital divide, creating omitted variable bias.

Therefore, this study re-matched the explanatory variables with a one-period lag and conducted the test. The results are reported in Column 3 of Table 4, revealing that the one-period lagged digital divide index still exacerbates current healthcare consumption inequality among residents, confirming that the baseline regression carries no risk of reverse causality.

### 4.4 Heterogeneity test

China continues to face challenges of uneven and inadequate development, a pattern that also persists in the healthcare consumption sector. This inequality manifests not only at the regional level but also at the individual level. Therefore, this study examines heterogeneous effects across two dimensions: region and individual characteristics.

#### 4.4.1 Regional level

Regional development imbalances persist in China, with a strong positive correlation between regional economic development and digital advancement (48). Therefore, this study examines heterogeneity along two dimensions: urban-rural differences and regional economic development levels. Two dummy variables (Urb, Eco) are constructed and interacted with the core explanatory variable, Divide, to estimate heterogeneous effects (Divide^*^Urb and Divide^*^Eco). The results, presented in Table 5, indicate that the impact of the digital divide on healthcare consumption inequality is significantly weaker among residents with rural household registration and those living in economically underdeveloped regions.

**Table 5:** Results of heterogeneity test.

**Variable**	**(1)**	**(2)**	**(3)**	**(4)**
	**Hc_inequality**			
Divide	0.0399^***^	0.0372^***^	0.1060^***^	0.0882^***^
	(0.0048)	(0.0039)	(0.0101)	(0.0085)
Divide^*^Urb	0.0520^***^			
	(0.0088)			
Urb	−0.0619^***^			
	(0.0080)			
Divide^*^Eco		0.0341^***^		
		(0.0074)		
Eco		−0.0174^***^		
		(0.0059)		
Divide^*^Edu			−0.0703^***^	
			(0.0084)	
Edu			0.0539^***^	
			(0.0065)	
Divide^*^Inc				−0.0714^***^
				(0.0100)
Inc				0.0509^***^
				(0.0066)
Controls	YES	YES	YES	YES
Area FE	YES	YES	YES	YES
Year FE	YES	YES	YES	YES
Obs	35,970	35,970	35,970	35,970
Adj R^2^	0.1224	0.1215	0.1262	0.1270

^*^, ^**^, ^***^ indicates statistical significance at the 1%, 5%, 10% level. The values in parentheses represent the cluster standard errors, with the same notation applied throughout.

Possible reasons include the following. First, in recent years, the state has vigorously promoted digital initiatives in rural areas, such as the “5G for Every Township” project, leading to improved digital literacy among residents and gradual enhancement of regional digital infrastructure (50). Second, residents in these areas may still primarily rely on traditional healthcare consumption methods—such as in-person medical consultations and offline medication purchases—rendering them less vulnerable to the adverse effects of the digital divide (51). In contrast, residents in urban and economically developed regions have achieved a high degree of digitalization in healthcare consumption. However, those facing digital access or literacy barriers may be unable to fully benefit from these advancements, thereby exacerbating inequality in healthcare consumption (52).

Third, rural households have significantly smaller healthcare expenditures, which limits the extent to which the digital divide can exacerbate inequality in healthcare consumption. According to the National Bureau of Statistics, the average annual healthcare expenditure of rural households is 3,215 CNY—only 41.6% of the urban average of 7,728 CNY. Rural residents primarily utilize basic, low-cost healthcare services and exhibit limited demand for premium digital health services, which tend to drive consumption disparities in urban areas. Because the digital divide primarily affects access to high-cost digital health services, its impact on overall healthcare consumption inequality is consequently limited in rural contexts. In contrast, urban residents with higher healthcare expenditures frequently use digital tools to access costly specialty care. As a result, digital exclusion generates a pronounced gap in healthcare spending between urban residents with and without access to digital technologies.

#### 4.4.2 Individual level

The effect of the digital divide on healthcare consumption inequality also varies across individuals (53). As previously discussed, engaging in digital healthcare consumption requires digital literacy, such as the ability to navigate health-related apps, and access to digital technologies is strongly associated with household income. Therefore, this study examines heterogeneous impacts in terms of education level and household income.

Consistent with the previous section, interaction terms are constructed to examine heterogeneity. Individuals are classified based on high school completion, and a dummy variable Edu is created (1 = high school graduate or above, 0 = otherwise). The interaction term Divide^*^Edu is then included in the regression model. Results in Column (3) of Table 5 show that the effect of the digital divide on healthcare consumption inequality varies significantly by education level. Specifically, residents without a high school education are more severely affected by the digital divide (54).

Additionally, a binary income variable, Inc, was constructed based on median household income (1 = at or above median, 0 = below median). The interaction term Divide^*^Inc was then included in the regression model. Results in Column (4) of Table 5 indicate that the effect of the digital divide on healthcare consumption inequality is more pronounced among higher-income groups. A possible explanation is that the impact of digital literacy disparities depends on whether individuals have sufficient economic resources to convert these advantages into actual healthcare consumption. Higher-income individuals are better positioned to adopt digital health services; thus, when hindered by the digital divide, they experience greater unmet potential, which in turn exacerbates inequality in healthcare consumption (55).

### 4.5 Further analysis

To delve deeper into the underlying mechanisms through which the digital divide influences inequality in residents' healthcare consumption, we employ stepwise regression and KHB regression methods to conduct empirical analysis of the transmission mechanisms based on the theoretical framework established earlier.

We utilize the Kakwani index from Model (1) to construct the resident income inequality index (Inc_inequality) as a proxy variable for income inequality.

The intertemporal consumption model suggests that credit constraints can be classified into formal and informal types. Formal constraints are typically mitigated through borrowing from financial institutions such as banks, whereas individuals facing informal constraints often rely on risk-sharing within social networks, including family and friends. Thus, liquidity constraints (Liquidity_constraints) reflect formal credit constraints, whereas social capital (Social_capital) reflects informal constraints.

Drawing on Zeldes (56), we adopt “financial assets exceeding two months of permanent income” (Permanent income = Annual net income—Transfer income and other income) as a proxy for liquidity constraints (Financial assets exceeding two months of permanent income indicate no liquidity constraints, coded as 0; otherwise coded as 1). Second, social gifting and other expenditures serve as crucial means for maintaining social capital. Drawing on Yi Xingjian (57), we use “whether social gifting expenditures occurred” as a proxy for social capital (occurrence of expenditures is coded as 1, otherwise as 0).

#### 4.5.1 Stepwise regression analysis

Table 6 reports the mediation effect results based on stepwise regression. Columns (1) and (2) show that income inequality acts as a significant mediator in the relationship between the digital divide and healthcare consumption inequality. Specifically, the two dimensions of the digital divide—accessibility to digital technologies (e.g., mobile internet/computer ownership) and usage of digital technologies (e.g., frequency of internet use for health-related activities) jointly exacerbate income inequality: accessibility barriers limit access to high-skill jobs and digital financial services, while usage barriers reduce the ability to optimize income-generating opportunities. This pathway is plausible because income is a key determinant of healthcare consumption; as the dual-dimensional digital divide widens income gaps, it consequently amplifies disparities in healthcare consumption.

**Table 6:** Results of the mediational effect test.

**Variable**	**(1)**	**(2)**	**(3)**	**(4)**	**(5)**	**(6)**
	**Inc_inequality**	**Hc_inequality**	**Liquidity_constraints**	**Hc_inequality**	**Social_capital**	**Hc_inequality**
Divide	0.0323^***^	0.0495^***^	0.1270^***^	0.0498^***^	−0.0378^***^	0.0520^***^
	(0.0028)	(0.0049)	(0.0103)	(0.0050)	(0.0065)	(0.0021)
Inc_inequality		0.0926^***^				
		(0.0163)				
Liquidity_constraints				0.0215^***^		
				(0.0021)		
Social_capital						−0.0132^***^
						(0.0047)
Controls	YES	YES	YES	YES	YES	YES
Area FE	YES	YES	YES	YES	YES	YES
Year FE	YES	YES	YES	YES	YES	YES
Obs	35,970	35,970	34,453	34,453	35,754	35,754
Adj R^2^	0.8083	0.1238	0.1305	0.1238	0.0975	0.1214

Due to questionnaire responses, the number of observations has changed. ^*^, ^**^, ^***^ indicates statistical significance at the 1%, 5%, 10% level. The values in parentheses represent the cluster standard errors, with the same notation applied throughout.

Columns (3)–(6) present the mediation results for credit constraints. The results suggest that both formal and informal credit constraints serve as significant mediating pathways through which the digital divide affects healthcare consumption inequality. This may occur because digitally disadvantaged individuals are often excluded from digital technologies and digital financial services, which limits their access to credit information and online lending platforms. As a result, they are more likely to face formal credit constraints. On the other hand, the rise of digital technology has transformed patterns of social interaction. The digital divide reduces social capital among individuals who lack access to or proficiency with social media platforms, thereby increasing their vulnerability to informal credit constraints (58). Both factors contribute to the emergence of inequality in healthcare consumption among residents.

#### 4.5.2 Mediating effect contribution rate testing

To assess the relative contributions of multiple mediating variables to the effect of the digital divide on healthcare consumption inequality, we apply the KHB method to decompose the indirect effects. The results are reported in Table 7.

**Table 7:** Mediation effect contribution rate.

**Variable**	**Inc_inequality**	**Liquidity_constraints**	**Social_capital**
Indirect effects	0.0029	0.0025	0.0002
P_Diff	51.7300	44.7300	3.5400
P_Reduce	5.9900	5.1800	0.4100

The results indicate that the mediation effects of variables Inc_inequality, Liquidity_constraints, and Social_capital are 0.0029, 0.0025, and 0.0002, respectively. The sum of these mediation effects is 0.0056, with contribution rates of 48.37%, 43.99%, and 7.64% for the three variables. These mediating variables explain 5.43%, 4.94%, and 0.86% of the total effect, respectively, yielding a total mediated proportion of 11.23%. Thus, the mediating contribution rates of the three variables, from highest to lowest, were: Inc_inequality, Liquidity_constraints, and Social_capital. This indicates that, among the mediating pathways, income inequality plays the most important role in transmitting the effect of the digital divide to healthcare consumption inequality, followed by formal credit constraints, with informal credit constraints having a relatively minor impact.

## 5 Discussion

This study confirms that the digital divide significantly exacerbates inequality in healthcare consumption, a conclusion that complements and extends the existing understanding of “technological inclusivity” in the digital economy era. Previous research has primarily emphasized the enabling effects of digital technology on healthcare consumption, such as improved service accessibility through online health platforms and more efficient resource allocation via digital systems. However, this study reveals exclusionary phenomena during the process of technological dissemination: the digital divide excludes the older adult, rural residents, and groups with low digital literacy from the benefits of digital healthcare through three dimensions—access, usage, and content—thereby creating inequalities in healthcare consumption. This finding aligns with the theoretical perspective that the digital divide exacerbates socioeconomic imbalances. Furthermore, by concentrating on healthcare consumption, a core dimension of household welfare, this study fills a critical gap in research on how the digital divide generates inequities in essential service consumption.

From a practical perspective, the research findings offer new insights into the causes of inequality in healthcare consumption in China. As per capita healthcare expenditure among Chinese residents has risen from CNY 1,902 in 2018 to an estimated CNY 2,547 in 2024, the coexistence of growing consumption levels and deepening inequality has become increasingly apparent. This study highlights that the digital divide is a key driver of this phenomenon: on one hand, digital healthcare services have become an important means for urban high-income groups to optimize their healthcare consumption; on the other hand, digitally disadvantaged groups continue to rely on traditional offline channels that are resource-unequal and cost-prohibitive, with the gap between the two groups continuing to widen.

This study validates the mediating role of income inequality and credit constraints in the impact of the digital divide on healthcare consumption inequality. This mechanism aligns closely with established theories of consumption behavior, which emphasize the role of income and access to credit in shaping consumption patterns. It also reflects the structural characteristics of China's evolving digital economy, where technological advancement coexists with persistent social disparities. Globally, the digital divide exacerbates healthcare consumption inequality across major developing economies, though its mechanisms vary by context. Compared with India and Brazil—both grappling with rural–urban divides and digital health transitions—China shares core challenges but exhibits distinct dynamics. All three countries see digital exclusion widen healthcare spending gaps, yet the intensity differs. In India, weak rural connectivity and linguistic barriers intensify inequality. In Brazil, digital tools are concentrated in private facilities due to healthcare privatization. China's unique pathway lies in how credit constraints, especially limited access to informal finance shaped by social capital among rural populations, mediate the link between digital exclusion and consumption inequality. These differences highlight that digital inclusion policies must reflect local structural conditions. China's focus on expanding rural digital infrastructure and delivering targeted digital literacy training offers practical lessons for other developing nations seeking to advance digital health equitably.

Regarding the mediating role of income inequality, this study finds that the digital divide indirectly exacerbates healthcare consumption inequality by increasing income disparities—a finding consistent with Keynes's absolute income hypothesis, which posits that consumption is primarily determined by current income levels. Further analysis highlights the skill-biased nature of the digital economy as a key mechanism: individuals with strong digital skills are more likely to obtain high-skill, high-income jobs, whereas those who are digitally disadvantaged encounter constrained labor market opportunities and stagnant income growth. The growing income gap directly translates into unequal access to and utilization of healthcare services. This mechanism is supported by empirical evidence from the CFPS: a one-unit increase in the digital divide index is associated with a 0.0323-unit rise in the income inequality index (*p* < 0.01), holding other factors constant. Income inequality further intensifies structural relative deprivation in healthcare access, reinforcing a self-reinforcing cycle of digital exclusion, income polarization, and consumption inequality.

Regarding the mediating role of credit constraints, this study reveals that the digital divide affects healthcare consumption through two parallel pathways: by intensifying both formal and informal credit constraints. This finding is consistent with the life cycle hypothesis, which emphasizes consumption smoothing through access to credit. From the perspective of formal credit, digitally disadvantaged groups face significant barriers in accessing fintech-enabled financial services due to low digital literacy and limited digital footprints. As a result, their probability of experiencing liquidity constraints increases by 12.70% (*p* < 0.01). In the realm of informal credit, the digital transformation of social interactions has altered how individuals maintain social networks. The digital divide hinders vulnerable populations, such as the older adult, from sustaining social capital through digital platforms, thereby reducing their participation in social exchanges and limiting access to informal lending sources. Given the high reliance of healthcare consumption on credit, individuals facing these constraints are often forced to reduce or forgo medical expenditures. This behavior further widens inequality in healthcare access and utilization.

The findings highlight significant regional and individual heterogeneity, providing actionable insights for tailored policy interventions to reduce the digital divide. They further underscore the disparities in the development of China's digital economy and healthcare consumption across different regions and demographic groups.

With respect to regional heterogeneity, the findings reveal a counterintuitive pattern: individual healthcare consumption inequality in rural and economically underdeveloped areas are less affected by the digital divide than in urban and high-income regions. This can be attributed to two key mechanisms. First, China's digital infrastructure initiatives, such as the “5G for Every Village” program, have yielded substantial progress, greatly enhancing digital connectivity in rural areas. By 2024, rural broadband penetration had exceeded 90%, substantially narrowing the digital divide at the access level. Second, healthcare consumption in rural and economically disadvantaged regions remains predominantly offline and less dependent on digital platforms, making these populations less vulnerable to digital exclusion. In contrast, in urban and high-income regions, digital healthcare has become the dominant mode of service delivery. Consequently, the digital divide directly limits access to high-quality care for disadvantaged groups, thereby deepening healthcare consumption inequality. These findings suggest that future digital infrastructure policies should shift focus from universal coverage to quality enhancement, and that targeted support programs should be implemented for digitally disadvantaged urban populations to prevent the emergence of intra-urban digital divides.

In analyzing individual heterogeneity, the study finds that the impact of the digital divide on healthcare consumption inequality varies by educational attainment and income level. First, individuals with a high school education or higher are less vulnerable to the digital divide. This is primarily because higher educational attainment is associated with stronger digital literacy. They exhibit greater ability to filter information and use digital tools effectively. In contrast, individuals with lower educational attainment often struggle to access and utilize digital healthcare resources due to limited digital literacy, thereby widening healthcare consumption disparities. Second, higher-income individuals are disproportionately affected by the digital divide despite their greater financial resources. This pattern likely arises from the tiered nature of digital healthcare services. High-income individuals often seek higher-quality care, but premium digital healthcare options typically come with higher fees. As a result, disparities in access to these services generate inequality even within high-income populations.

This study provides key empirical evidence for understanding healthcare consumption inequality in the digital age and offers corresponding policy recommendations. First, healthcare authorities should deepen the decentralization of digital healthcare resources by leveraging existing service networks, such as primary healthcare centers and community health service centers. Specific measures include equipping primary care facilities with telemedicine consultation systems and providing one-on-one digital health service guidance for older adult populations. Additionally, specialized digital healthcare advancement plans should be developed for rural and underdeveloped regions, with priority given to implementing essential digital services such as online appointment booking, electronic health record access, and chronic disease management platforms. By digitizing healthcare delivery scenarios, this initiative aims to help individuals with limited digital literacy access medical resources more easily and reduce disparities in healthcare consumption. Second, government digital infrastructure agencies should prioritize investment in rural and underdeveloped areas, establishing clear targets for network coverage and promoting affordable, high-speed internet access. They should also develop age-friendly and user-friendly digital healthcare applications, strengthen regulatory oversight to ensure data security, and establish a shared digital skills training platform. This platform should offer customized courses and support blended online and offline training programs. Such efforts would address the digital divide from three complementary dimensions: infrastructure, tools, and capabilities. Third, residents are encouraged to actively participate in digital literacy programs, learning to use digital healthcare services such as online appointment systems and medical information portals through offline workshops or mobile learning applications. They should learn to identify authoritative platforms to avoid misinformation and plan their healthcare consumption more efficiently. Participation in community health lectures and digital healthcare experience events is also recommended to foster integration into the community's digital health ecosystem, thereby enhancing both digital competence and the efficiency of healthcare resource utilization. Finally, strengthen cross-regional digital health cooperation: Developed eastern regions and leading digital health cities should form long-term partnerships with underdeveloped rural areas in central and western China, co-developing interoperable infrastructure, sharing telemedicine resources through consortia, and jointly training professionals via mentorship and exchanges. Such efforts can overcome geographic inequities and empower underserved regions to effectively use digital health tools.

Although this study has clearly established the relationship between the digital divide and inequality in healthcare consumption, it still has limitations that can provide directions for future research improvements: First, in terms of the measurement dimensions of the digital divide, this study only covers two dimensions—access and usage—and does not include other factors. Future research could incorporate the newly added “digital security perception” items in the CFPS to improve the indicator system. Second, in terms of the timeliness of the research sample, the data used in this study is up to 2022, while China's digital healthcare policies and consumption patterns have undergone new changes since 2023. Future research could incorporate the latest data to examine the moderating effect of policy interventions on the relationship between the digital divide and healthcare consumption inequality.

Although this study has clearly established the relationship between the digital divide and healthcare consumption inequality, it has certain limitations that suggest avenues for future research. First, regarding the measurement of the digital divide, this study focuses only on access and usage, excluding other important dimensions such as digital skills, literacy, and security. Future studies could incorporate the newly added “digital security perception” items in the CFPS to enhance the comprehensiveness of the measurement framework. Second, with respect to sample timeliness, the data used in this study extend through 2022, whereas China's digital healthcare policies and consumption patterns have evolved significantly since 2023. Future research should incorporate more recent data to assess how policy interventions moderate the relationship between the digital divide and healthcare consumption inequality.

## 6 Conclusion

Healthcare consumption inequality is a key manifestation of the principal contradiction in Chinese society. Alleviating this inequality is essential for improving people's quality of life and achieving common prosperity. This study uses multi-wave China Family Panel Survey data to empirically examine the impact of the digital divide on healthcare consumption inequality, as well as its underlying mechanisms and heterogeneity. The main conclusions are as follows: (1) The digital divide significantly exacerbates residents' healthcare consumption inequality; (2) The impact of the digital divide on inequality in residents' healthcare consumption varies across individuals and regions; and (3) The digital divide intensifies residents' healthcare consumption inequality by widening income gaps and reinforcing credit constraints. This study empirically demonstrates the relationship between the digital divide and healthcare consumption inequality using existing data, proposing corresponding policy recommendations to help prevent and address healthcare consumption inequality in the digital era.

## Data Availability

Publicly available datasets were analyzed in this study. This data can be found here: CFPS,www.isss.pku.edu.cn/cfps.
